# Facial soft tissue changes in adolescent patients treated with three different functional appliances: a randomized clinical trial

**DOI:** 10.1590/2177-6709.29.5.e242440.oar

**Published:** 2024-10-07

**Authors:** Nathália Moraes Carvalho Barreto BRANDÃO, Nathália Barbosa PALOMARES, Tatiana LIMA, Cátia Cardoso Abdo QUINTÃO, Klaus Barretto LOPES, José Augusto Mendes MIGUEL

**Affiliations:** 1 State University of Rio de Janeiro, School of Dentistry, Department of Orthodontics (Rio de Janeiro/RJ, Brazil).

**Keywords:** Angle Class II malocclusion, Functional orthodontic appliances, Randomized clinical trials, Má oclusão Classe II de Angle, Aparelhos ortodônticos funcionais, Ensaios clínicos randomizados

## Abstract

**Introduction:**

Patients with Class II, division 1 malocclusion generally seek treatment to improve facial esthetics. Therefore, the orthodontist needs to know the changes in the soft profile produced by functional appliances.

**Objective:**

This study evaluated the soft tissue profile changes in patients treated during the peak of the pubertal growth spurt.

**Methods:**

Thirty selected patients were randomized into three treatment groups: Twin Block (TB), Herbst with dental anchorage (HDA), and Herbst with skeletal anchorage (HSA). All patients had computed tomographic images: pretreatment (T1) and after 12 months of active treatment (T2). Twenty-four soft tissue cephalometric measures were analyzed. The normality of all data was assessed by the Shapiro-Wilk test. Intragroup comparisons were analyzed using the *t*-paired test; the inter-group comparisons were determined through ANOVA and the *post-hoc* Tukey test.

**Results:**

At T1, no significant differences were observed between groups. At T2, in the intragroup comparison, facial soft tissue changes were statistically significant in the three groups for the lower lip, sulcus inferioris, facial soft tissue convexity in HDA group and TB group, and H angle in HDA group and HSA group, and soft tissue pogonium in TB group. In the inter-group comparison, no statistically significant differences were observed.

**Conclusion:**

It can be concluded that there were significant changes in soft tissue measurements that benefited Class II, division 1 patient’s facial profile treated with the functional appliances Twin Block, Herbst, and Herbst with skeletal anchorage. Nevertheless, no significant differences were detected among the effects obtained by the three treatment protocols.

## RESUMO

**Introdução:**

Pacientes portadores de má oclusão de Classe II, divisão 1, geralmente buscam tratamento para obter melhora da estética facial. Logo, é essencial que o ortodontista conheça as alterações do perfil mole associadas a uma das principais modalidades de tratamento dessa má oclusão: os aparelhos propulsores mandibulares.

**Objetivo:**

O presente estudo avaliou as alterações nos tecidos moles do perfil facial em pacientes tratados durante o pico do surto de crescimento puberal com aparelhos propulsores mandibulares removível, fixo ou fixo associado à ancoragem esquelética.

**Métodos:**

Trinta pacientes foram randomizados em três grupos: Twin Block (TB), Herbst com ancoragem dentária (HAD) e Herbst com ancoragem esquelética (HAE). Os pacientes realizaram tomografias computadorizadas pré-tratamento (T1) e após 12 meses de tratamento (T2). Foram analisadas 24 medidas cefalométricas em tecidos moles. A normalidade dos dados foi avaliada pelo teste de Shapiro-Wilk, as comparações intragrupo foram feitas por meio do teste *t* pareado; as comparações intergrupo, feitas com ANOVA e o teste *post-hoc* de Tukey.

**Resultados:**

Em T1, não foram observadas diferenças significativas entre os grupos, confirmando a homogeneidade da amostra. Na comparação intragrupos, foram detectadas alterações estatisticamente significativas nos três grupos, na convexidade facial de tecido mole, sulcus inferioris e pogônio mole; e na comparação intergrupo não foram observadas diferenças estatisticamente significativas.

**Conclusão:**

Após o tratamento com os aparelhos ortopédicos, foram detectadas alterações significativas que beneficiaram os tecidos moles da face de pacientes Classe II, divisão 1. Porém, não foram detectadas diferenças significativas nos efeitos obtidos entre os três tipos de aparelhos.

## INTRODUCTION

The improvement of dentofacial aesthetics is one of the main objectives of orthodontic treatment, both from the point of view of orthodontists and patients.^1^ ^-^ ^4^ Although not all the parameters involved in the ideal facial esthetics are fully known, research indicates that there is a preference for a straight profile.^5^ ^,^ ^6^ The convex profile, which is considered less pleasant from an aesthetic point of view, is generally associated with Angle Class II malocclusion,^7^ in particular the division 1, characterized by an accentuated projection of the maxillary incisors. The soft tissue characteristics of these patients involve a high degree of facial convexity, with a diminished mentolabial angle, labial incompetence, receding lower lip and chin, and short chin-to-neck length.^8^

Orthopedic control during the pubertal growth spurt is the most effective, stable, and least deleterious treatment for Class II, division 1 malocclusion.^9^ ^-^ ^14^ As the studies have shown that most patients with this malocclusion present mandibular retrognathism, various mandibular propulsion devices have been developed, such as: Bionator, Activator, MARA, Functional Regulator, Herbst, Twin Block, Jasper Jumper, Forsus, PowerScope. Among these, the most notable are the Twin Block (removable) and Herbst (fixed) devices, due to their high efficiency, demonstrated by several clinical trials and systematic reviews.

The efficacy of fixed and removable functional appliances in the treatment of patients during growth with skeletal Class II was analyzed by a systematic review.^15^ Of the 989 articles identified, only two randomized clinical trials and two controlled clinical trials were included. Analysis of the cephalometric data showed that all the appliances corrected the patient’s dental overjet due to a combination of skeletal and dental effects. However, only one included study evaluated changes in soft tissue, which demonstrated that Herbst and Twin Block devices induced significant and similar favorable changes in the patient profile. The authors observed that this result was surprising, as it did not corroborate the results of two previous clinical trials, which failed to demonstrate detectable changes in the soft-profile silhouettes of children treated with removable functional appliances. It was concluded that there is still insufficient evidence to compare the efficacy of fixed and removable functional appliances about the effects obtained in the soft profile.

Other study compared the effects of Herbst and Twin Block appliances on the soft tissues of patients treated at the peak of the pubertal growth spurt,^16^ observing that the effects obtained by the devices were similar, with significant changes in soft tissue, compared to the control group, concluding that greater advancement of soft pogonium and lower lip was observed in patients treated with Twin Block than those treated with Herbst appliance.

The effects of fixed functional appliances on Class II treatment were evaluated in other study,^17^ concluding that there was a significant improvement in the profile of the treated patients.

Since 2004, the use of skeletal anchorage associated with fixed functional appliances has been suggested to try to potentiate skeletal changes.^18^ ^-^ ^21^ One study compared the skeletal and dentoalveolar effects of using fixed mandibular protractors with and without skeletal anchorage.^22^ The low level of available evidence suggested that the association of skeletal anchorage did not induce additional skeletal changes. No analysis of changes in the soft profile was performed.

Although one of the main objectives of treatment with mandibular protractors is to obtain improvement in the soft profile and consequently in patients’ facial aesthetics, literature presents scarce studies, with controversial results on the effects of this therapy on the soft tissues of the facial profile.^16^ ^,^ ^23^ ^,^ ^24^ In general, studies focus only on skeletal and dental effects. Therefore, further studies are needed to assess soft tissue changes.

## OBJECTIVES

The objective of the present study was to compare the soft profile changes in patients treated with removable appliance (Twin Block), fixed appliance (Herbst), or fixed appliance with skeletal anchorage (Herbst with skeletal anchorage).

## METHODS

### TRIAL DESIGN

This was a randomized clinical trial, with three parallel groups, enrolled in ClinicalTrials.gov (protocol ID: NCT0241812). The Consort Statement standards were followed.^25^

### PARTICIPANTS

#### ELIGIBILITY CRITERIA

The inclusion criteria were:

1) Children aged 10 to 14 years.
2) Angle Class II, division 1 malocclusion.
3) Complete permanent dentition up to the first molars.
4) Convex profile.
5) Overjet ≥ 6 mm.
6) Skeletal maturation of the cervical vertebrae in stages CS3 and CS4, according to Baccetti et al.^26^
7) Carpal skeletal maturation in the FPcap and FMcap stages, according to Mercadante.^27^

Patients were excluded if they presented:

1) History of previous orthodontic treatment.
2) Cleft lip and/or palate, craniofacial syndromes, or congenital diseases.
3) Dental agenesis.
4) Supernumerary teeth.
5) Signs/symptoms of temporomandibular dysfunction.
6) Poor oral hygiene.

All the included patients were informed of the characteristics and objectives of the research, and signed an informed consent form. The study was approved by the Ethics and Research Committee of the Rio de Janeiro State University (CAAE: 46731015.6.0000.5259), linked to the Brazil Platform in June / 2015.

### SETTINGS AND LOCATIONS WHERE THE DATA WERE COLLECTED

Patients who met the inclusion criteria were selected from four different places: The Rio de Janeiro State University Dental School and three public schools (Application Institute Fernando Rodrigues da Silveira, Municipal School República Argentina, and the Municipal School Equador). All orthodontic treatments were performed at the Orthodontic clinic of the Rio de Janeiro State University (Brazil).

### INTERVENTIONS

After the patient’s selection and signing of the written informed consent, complete orthodontic documentation was performed, consisting of facial and intraoral photographs, plaster models, and cone-beam computed tomography. All three groups were treated for twelve months. After this treatment period, all appliances were removed, and new complete documentation was performed.

**Figure 1:**
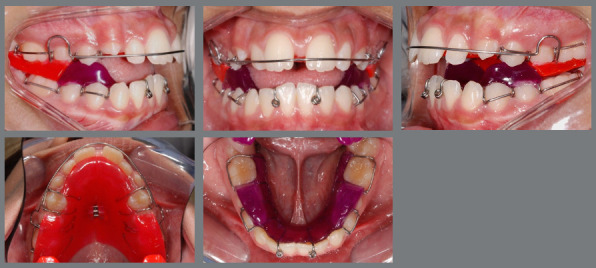
Intraoral photographs illustrating the TB group, treated with the Twin Block removable appliance.

The TB group was treated with the Twin Block removable device, consisting of retention clamps and two acrylic resin bite blocks (upper and lower) according to the Clark protocol^28^ (Fig 1). Patients were motivated to use it full-time, except during meals and hygiene.

**Figure 2:**
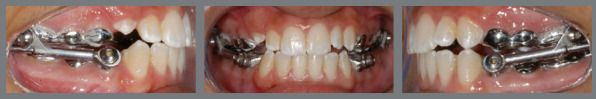
Intraoral photographs illustrating the HDA group, treated with the Herbst fixed appliance.

The HDA group was treated with the splinted Herbst fixed appliance, made from a chromium-cobalt alloy (Fig 2). It was cemented to the permanent upper and lower first molars, first and second premolars, as described by Pancherz and Ruf.^29^

**Figure 3:**
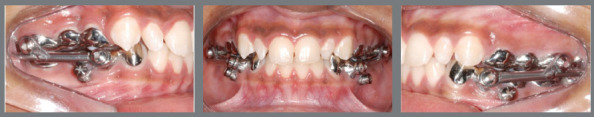
Intraoral photographs illustrating the HSA group, treated with the Herbst fixed appliance with skeletal anchorage.

The HSA group was treated with the Herbst appliance, using the same design and manufacturing process as the HDA Group. Additionally, two orthodontic mini-screws (2mm in diameter and 10mm in length, Neodent, Curitiba, Brazil) were installed in the alveolar bone crest region, between the roots of the first and second mandibular premolars, bilaterally. Orthodontic wire for ligature (0.012-in thickness) was installed between the head of each mini-implant, and a bracket was welded to the appliance structure (Fig. 3).

After the installation, the patients were followed-up monthly. Computed tomography (CT) scans were obtained pre-treatment (T1) and after twelve months of treatment (T2). All CT scans were digitally traced by a single operator previously calibrated, with blinding for the type of treatment performed, using Dolphin Imaging v. 11.7 software (Dolphin Imaging and Management Systems, Chatsworth, CA, USA).

### OUTCOME MEASURES

**Figure 4:**
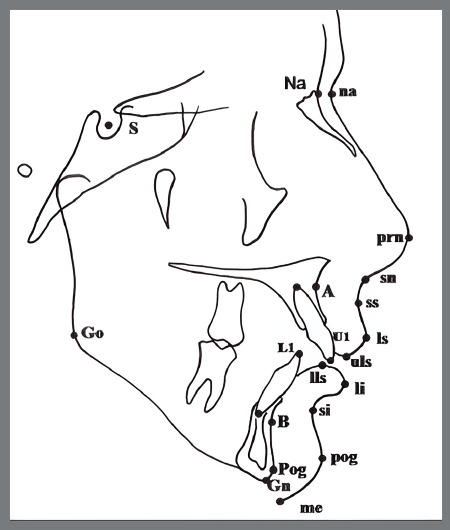
Landmarks used in the study.

**Figure 5:**
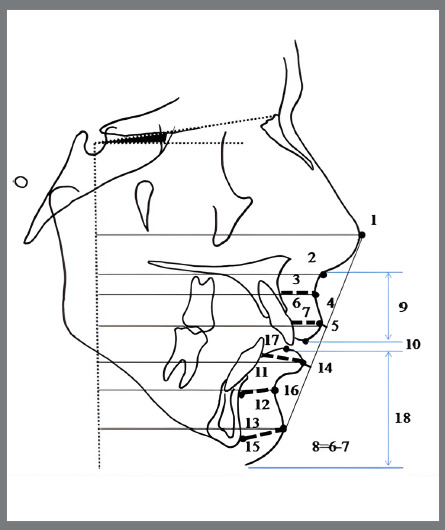
Soft tissue linear measurements, according to the Appendix 1.

Primary and secondary outcome measures were obtained from the cephalometric analysis used by Baysal and Uysal.^16^ This analysis was originally developed by Illing et al.^30^ to compare the dental, skeletal and soft-profile effects of Bass, Bionator, and Twin Block functional appliances. The analysis covers 24 measurements, being 6 angular and 18 linear, which were evaluated in each cephalometric radiograph (Appendix 1). The points used are shown in Figure 4. A horizontal reference line was constructed 7º below the Sella-Nasion line, and a vertical reference line was drawn perpendicularly to this horizontal reference line, passing through the Sella. Linear measurements of soft tissues were drawn according to this vertical reference line (Fig. 5). For precision, the soft tissue angular measures were considered as primary outcome measures; and soft tissue linear measures, as secondary outcomes, as follows:

### PRIMARY OUTCOME MEASURES

Soft tissue angular measures:

» Position of the soft tissue Na-Prn-Pog; Na-Sn-Pog; H angle; Nasolabial angle; Mentolabial angle.

### SECONDARY OUTCOME MEASURES

Soft tissue linear measures:

» Maxilla: VRL-prn; VRL-sn; VRL-ss; VRL-ls; E-ls; Basic upper lip thickness; Upper lip thickness; Lip strain; sn-uls; Interlabial gap.

» Mandible: VRL-li; VRL-si, E-li; VRL-pog; Pog-Pog’; Si-B; Lower lip thickness; lls-me.

### SAMPLE SIZE

The sample size calculation was performed using the software developed by Harvard University, which can be found on the following website:

*http://hedwig.mgh.harvard.edu/sample_size/js/js_parallel_quant.html.* The level of significance was set at 0.05, with a power of 0.8 and a minimal detectable difference in means of 1 standard deviation. A total of 28 patients were needed.

### RANDOMIZATION

The sequence generation was performed by block randomization using a list generated by randomization.com software. The allocation sequence was concealed in opaque envelopes numbered with treatment allocation. The implementation of randomization (generation and storage of the list, envelopes, allocation, concealment, and signature of treatments) was performed by the secretary of the Department of Orthodontics of the Faculty of Dentistry of the Rio de Janeiro State University. Blinding was performed only for the analysis of the results. 

### STATISTICAL METHODS

All data analyses were performed in the SPSS program (version 12.0, Chicago, Illinois, USA). The Shapiro-Wilk normality test was applied to the data. The normality of all data was detected, and parametric tests were selected. Arithmetic averages and standard deviations were calculated for each measure. Intragroup comparisons were analyzed using the *t*-paired test, and intergroup comparisons were determined by one-way analysis of variance (ANOVA) and possible *post-hoc* multiple comparisons using the Tukey’s Range Test (HSD).

Ten CT scans of patients not belonging to the sample were randomly selected to determine the errors of the method associated with cephalometric measures. Their tracings were repeated two weeks after the first measurements. The intraclass correlation index was calculated between the first and second measurements, to evaluate the error of the method. Excellent reproducibility (ICC = 0.92; 95% CI 0.88-0.94) was obtained, and the examiner started the cephalometric tracings.

Patients who left the study early were not evaluated, and therefore the intention-to-treat analysis (ITT) was not performed. A “per protocol” analysis was performed instead. As a result, only the patients who completed the study were assessed.

## RESULTS

### PARTICIPANT FLOW AND NUMBERS ANALYZED

**Figure 6:**
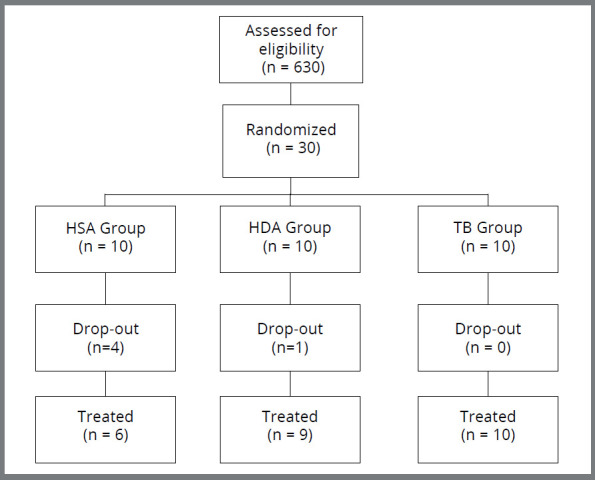
Flowchart of patients in study.

A total of 630 patients were assessed for eligibility. Five out of the thirty selected and randomized individuals dropped out of treatment (4 from the HSA group and 1 from the HDA group). The reasons for stopping treatment were related to the difficulty in adapting to the Herbst appliance at the beginning, mainly due to the presence of skeletal anchorage on miniscrews. All the patients from the TB group completed the treatment. Thus, 6 patients from the HSA group were treated, 9 from the HAD group, and 10 from the TB group. The participant flow can be seen in the Figure 6. 

### RECRUITMENT

Recruitment started in August 2015 and was completed in 2021. Patients were followed-up until 2023.

### BASELINE DATA

No relevant difference was observed in the patients’ gender and chronological age distribution (Table 1). No statistically relevant differences were detected among the groups, reassuring the sample’s homogeneity in T1 (Table 2). 

**Table 1:** Mean ages and gender distribution of the subjects.

	HDA	HSA	TB	Significance
AGE (years)	12.7 ± 1.18	11.8 ± 1.36	12.2 ± 0.80	NS
GENDER				
Female, n (%)	3 (33.3)	3 (50)	4 (40)	
Male, n (%)	6 (66.6)	3 (50)	6 (60)	

HDA = Herbst with dental anchorage. HSA = Herbst with skeletal anchorage. TB = Twin Block.

NS = non-significant

**Table 2:** Comparison of groups at baseline.

Measurements	HDA		HSA		TB		ANOVA
	Mean	SD	Mean	SD	Mean	SD	
SOFT TISSUE ANGULAR							
Na-Prn-Pog	128.8	4.72	129.8	2.60	128.9	2.91	0.856
Na-Sn-Pog	156.5	6.81	152.8	11.05	155.7	3.96	0.613
H angle	20.7	4.01	20.00	2.56	22.25	3.11	0.406
Nasolabial	34.33	6.42	31.56	5.96	31.66	3.07	0.469
Mentolabial	123.8	10.81	110.6	17.68	114.4	22.6	0.342
SOFT TISSUE LINEAR							
Maxillary							
VRL-prn	104.8	7.30	99.38	7.76	104.1	5.13	0.282
VRL-sn	91.83	6.61	88.86	10.89	91.19	4.74	0.729
VRL-ss	92.12	6.77	87.56	8.67	91.71	4.28	0.367
VRL-ls	95.88	8.09	90.26	8.60	95.82	4.86	0.264
E-ls	1.91	3.73	0.58	2.19	2.02	2.22	0.592
Basic upper lip thickness	25.43	2.59	21.80	1.72	24.57	2.23	0.019
Upper lip thickness	13.37	1.98	13.41	1.59	13.12	1.92	0.938
Lip strain	12.05	1.41	8.38	2.97	11.45	1.90	0.007
sn-uls	26.41	2.53	5.96	4.97	23.11	2.95	0.100
Interlabial gap	2.06	1.63	1.13	0.60	3.57	4.06	0.234
Mandibular							
VRL-li	89.94	9.04	84.60	8.37	88.83	5.84	0.416
VRL-si	79.24	8.30	75.46	8.55	78.63	7.67	0.658
E-li	3.74	4.29	3.08	2.07	3.49	3.06	0.934
VRL-pog	73.04	19.37	75.83	7.90	77.90	11.67	0.875
Pog-pog	13.64	1.52	14.50	2.29	14.12	3.14	0.800
Si-B	13.35	1.51	13.26	1.10	13.21	1.66	0.978
Lower lip thickness	16.57	2.58	16.76	2.27	16.71	2.08	0.986
lls-me	20.71	2.97	18.96	2.42	20.54	4.92	0.652

HDA= Herbst with dental anchorage. HSA= Herbst with skeletal anchorage. TB=Twin Block.

### OUTCOMES

#### Intragroup comparison T1-T2 (Table 3)

**Table 3:** Descriptive table of the values obtained for cephalometric measurements of soft tissue in T1 and T2 of the three groups treated.

Measurements	HDA group		t test	HSA group		t test	TB group		t test
	T1	T2		T1	T2		T1	T2	
	Mean (SD)	Mean (SD)		Mean (SD)	Mean (SD)		Mean (SD)	Mean (SD)	
SOFT TISSUE ANGULAR										
Na-Prn-Pog	128.8 (4.72)	130.26 (5.45)	0.005*	129.8 (2.60)	132.93 (3.73)	0.086	128.9 (2.91)	129.58 (3.58)	0.049*
Na-Sn-Pog	156.5 (6.81)	158.36 (6.38)	0.000*	152.8 (11.05)	160.88 (4.00)	0.681	155.7 (3.96)	156.98 (3.26)	0.025*
H angle	20.7 (4.01)	19.06 (3.74)	0.001*	20.00 (2.56)	16.73 (2.74)	0.038*	22.25 (3.11)	20.31 (3.34)	0.094
Nasolabial angle	34.33 (6.42)	33.96 (5.79)	0.017*	31.56 (5.96)	31.7 (4.68)	0.057	31.66 (3.07)	31.84 (4.31)	0.211
Mentolabial angle	123.8 (10.81)	126.84 (14.17)	0.088	110.6 (17.68)	122.5 (14.27)	0.169	114.4 (22.64)	119.56 (18.18)	0.074
SOFT TISSUE LINEAR										
Maxillary									
VRL-prn	104.8 (7.30)	105.00 (6.97)	0.028*	99.38 (7.76)	100.93 (9.09)	0.014*	104.1 (5.13)	106.15 (6.48)	0.000*
VRL-sn	91.83 (6.61)	91.76 (6.89)	0.020*	88.86 (10.89)	88.23 (9.64)	0.137	91.19 (4.74)	92.39 (5.57)	0.000*
VRL-ss	92.12 (6.77)	91.58 (7.05)	0.043*	87.56 (8.67)	87.78 (9.45)	0.013*	91.71 (4.28)	92.49 (5.09)	0.000*
VRL-ls	95.88 (8.09)	95.72 (7.53)	0.010*	90.26 (8.60)	91.38 (9.65)	0.014*	95.82 (4.86)	96.98 (5.61)	0.000*
E-ls	1.91 (3.73)	0.86 (2.96)	0.007*	0.58 (2.19)	-0.95 (1.64)	0.002*	2.02 (2.22)	0.91 (2.93)	0.004*
Basic upper lip thickness	25.43 (2.59)	24.45 (3.62)	0.001*	21.80 (1.72)	23.41 (3.27)	0.131	24.57 (2.23)	24.74 (2.96)	0.272
Upper lip thickness	13.37 (1.98)	13.68 (1.86)	0.053	13.41 (1.59)	14.50 (1.82)	0.465	13.12 (1.92)	15.14 (1.54)	0.276
Lip strain	12.05 (1.41)	10.76 (3.53)	0.286	8.38 (2.97)	8.91 (3.82)	0.112	11.45 (1.90)	9.60 (2.12)	0.55
sn-uls	26.41 (2.53)	25.78 (2.15)	0.45	25.96 (4.97)	24.23 (2.92)	0.737	23.11 (2.95)	23.93 (3.16)	0.019*
Interlabial gap	2.06 (1.63)	1.06 (0.94)	0.182	1.13 (0.60)	0.71 (0.84)	0.541	3.57 (4.06)	2.60 (2.83)	0.598
Mandibular									
VRL-li	89.94 (9.04)	91.36 (7.34)	0.037*	84.60 (8.37)	88.51 (10.95)	0.018*	88.83 (5.84)	91.38 (7.06)	0.000*
VRL-si	79.24 (8.30)	80.75 (6.96)	0.039*	75.46 (8.55)	78.50 (9.81)	0.040*	78.63 (7.67)	81.18 (8.20)	0.000*
E- li	3.74 (4.29)	3.51 (3.15)	0.000*	3.08 (2.07)	2.78 (3.14)	0.003*	3.49 (3.06)	4.11 (4.27)	0.001*
VRL-pog	73.04 (19.37)	79.06 (6.27)	0.583	75.83 (7.90)	79.33 (8.34)	0.066	77.90 (11.67)	79.72 (11.67)	0.000*
Pog-pog	13.64 (1.52)	13.65 (1.67)	0.226	14.50 (2.29)	14.9 (1.96)	0.016	14.12 (3.14)	15.72 (5.04)	0.038*
si-B	13.35 (1.51)	13.94 (1.97)	0.695	13.26 (1.10)	13.91 (1.37)	0.185	13.21 (1.66)	14.71 (3.14)	0.047*
Lower lip thickness	16.57 (2.58)	16.41 (2.78)	0.965	16.76 (2.27)	16.23 (2.63)	0.042	16.71 (2.08)	16.81 (3.35)	0.272
lls-me	20.71 (2.97)	21.57 (1.79)	0.438	18.96 (2.42)	19.71 (2.41)	0.345	20.54 (4.92)	21.17 (4.93)	0.022

*p < 0.05. HDA= Herbst with dental anchorage. HSA= Herbst with skeletal anchorage. TB=Twin Block.

» HDA Group. The convexity angles of the soft tissue, including the nose (Na-Prn-Pog) and excluding the nose (Na-Sn-Pg), were respectively increased by 1.46° and 1.86° (*p*<0.05). The nasolabial angle and the H angle decreased, respectively, 0.37° and 1.64° (*p*<0.05). In the maxilla, the measures VRL-ss (0.54mm), VRL-ls (0.16mm), E-ls (-1.05mm), and basic upper lip thickness (-0.98mm) were significantly reduced (*p*<0.05). The lower lip (+1.42mm) and the inferior groove (+0.59mm) were moved to anterior. The distance from the lower lip to the E plane decreased 0.23mm.

» HSA Group. The soft tissue convexity angles were increased by 3.13° and 8.0°, respectively, but these changes were not statistically significant (*p* = 0.086 and 0.68, respectively). The H angle decreased 3.27° (*p*<0.05). The upper lip (+1.12 mm), upper groove (+0.22 mm), and nose (+1.55 mm) were significantly moved to anterior in relation to the VRL (*p*<0.05). The distance between the upper lip and the E plane decreased by 1.53 mm (*p*<0.05). A significant increase in VRL-li (3.91mm), VRL-si (3.04mm) and Pog-pog (0.40mm) (*p* <0.05) was observed after treatment. 

» TB Group. As in the other groups, there was an increase in the soft tissue convexity angle including the nose (0.68°) and excluding the nose (1.28°), in a statistically significant way (*p*<0.05). The nasolabial angle increased 0.18° and the H angle decreased 1.94°, but these last two were not statistically significant (*p* = 0.094). All VRL-related measures had statistically significant changes (*p*<0.05) and presented increase: VRL-prn (2.05mm); VRL-sn (1.92mm); VRL-ss (0.78mm); VRL-ls (0.86mm); VRL-li (2.55mm); VRL-si (2.55mm); VRL-pog (1.82mm). The distance from the upper lip to the E plane decreased 1.11mm, the upper lip length increased 0.82mm; the interlabial distance decreased 0.97mm. Except for the lower lip thickness, which increased 0.10mm, all other soft tissue measurements had statistically significant changes (*p*<0.05) with the treatment. 

#### Intergroup comparison (Table 4)

**Table 4:** Comparison of mean differences between treated groups.

Measurements	HDA	HSA	TB	ANOVA
	Mean (SD)	Mean (SD)	Mean (SD)	
SOFT TISSUE ANGULAR				
Na-Prn-Pog	1.43 (2.98)	3.10 (2.47)	0.64 (2.85)	0.260
Na-Sn-Pog	1.86 (1.96)	8.05 (12.54)	1.27 (2.88)	0.115
H angle	-1.71 (1.76)	-3.26 (1.53)	-1.94 (3.04)	0.427
Nasolabial angle	-0.36 (4.24)	0.13 (3.59)	0.18 (4.07)	0.952
Mentolabial angle	2.96 (11.57)	22.9 (37.35)	5.08 (18.95)	0.225
SOFT TISSUE LINEAR				
Maxillary				
VRL-prn	0.17 (5.32)	1.55 (3.97)	2.14 (2.14)	0.561
VRL-sn	-0.06 (4.78)	-0.63 (8.29)	1.20 (1.75)	0.752
VRL-ss	-0.53 (5.51)	0.21 (4.03)	0.78 (1.97)	0.781
VRL-ls	-0.16 (4.99)	1.11 (4.18)	1.16 (2.16)	0.722
E-ls	-1.04 (2.12)	-1.53 (0.77)	-1.11 (1.68)	0.850
Basic upper lip thickness	-0.97 (2.73)	1.08 (1.92)	0.17 (3.12)	0.243
Upper lip thickness	0.31 (0.91)	1.61 (2.43)	2.02 (1.94)	0.099
Lip strain	-1.28 (3.24)	0.53 (2.69)	-1.85 (2.52)	0.278
sn-uls	-0.62 (2.81)	-1.73 (5.43)	0.82 (2.29)	0.351
Interlabial gap	-1.00 (2.25)	-0.41 (0.87)	-0.97 (4.49)	0.931
Mandibular				
VRL-li	1.42 (6.56)	3.91 (5.19)	2.55 (2.56)	0.637
VRL-si	1.51 (6.13)	3.03 (5.45)	2.55 (3.40)	0.830
E-li	-0.23 (1.84)	-0.30 (1.31)	0.62 (2.12)	0.522
VRL-pog	1.65 (6.46)	3.50 (5.38)	1.82 (2.88)	0.757
Pog-pog	0.01 (1.68)	0.61 (1.05)	1.60 (3.79)	0.441
Si-B	0.58 (1.43)	0.65 (1.10)	1.50 (2.44)	0.515
Lower lip thickness	-0.16 (0.73)	-0.56 (1.48)	0.10 (3.19)	0.844
lls-me	0.86 (2.97)	0.75 (2.48)	0.63 (3.78)	0.987

HDA= Herbst with dental anchorage. HSA= Herbst with skeletal anchorage. TB=Twin Block.

It was noticed increase in the soft tissue convexity angles Na-Prn-Pog (HSA: 3.10°, HDA: 1.43°, TB: 0.64°, *p*= 0.260), Na-Sn-Pog (HSA: 8.05°, HDA: 1.86°, TB: 1.27°, *p*= 0.115) and mentolabial (HSA: 22.90°, TB: 5.08°; HDA: 2.96°; *p*= 0.225). The H angle decreased (HSA: 3.26°, TB: 1.94°, HDA: 1.71°, *p*= 0.427). In relation to the linear measurements, the tip of the nose suffered an increase in distance (TB: 2.14mm; HSA: 1.55mm; HDA: 0.17mm; *p*= 0.561). The interlabial distance presented a decrease (HDA: 1.00 mm; TB: 0.97 mm; HSA: 0.41 mm; *p*= 0.931), which indicates improvement in the labial sealing. All linear measures of soft tissue related to the maxilla and mandible were altered in such a way that benefited the patients’ profiles: VRL-li (HSA: +3.91mm; TB: +2.5mm; HDA: +1.42mm; *p* = 0.637 ); VRL-si (HSA: +3.03mm, TB: +2.55mm, HDA: +1.51mm, *p* = 0.830) and VRL-Pog (HSA: +5.38mm; TB: + 1.82mm; HDA: 1.65mm, *p* = 0.757); soft tissue thickness of the chin (TB: +1.60mm; HSA: +0.61mm; HDA: +0.01mm; *p*= 0.441); lower lip thickness at the lower groove level (TB: + 1.50mm; HSA: + 0.65mm; HDA: + 0.58mm, *p*= 0.515) and the lower lip length (HDA: +0.86mm; HSA: +0.75mm, TB: +0.63mm, *p*= 0.987). All of these changes demonstrated that treatment with the mandibular propulsion appliances induced protrusion in the position of the lower lip and the soft pogonium, and an increase in the length of the lower lip and the thickness of the lower lip. However, no significant differences were detected among the results obtained by the three groups. 

### HARMS

There was no relevant harm caused by the use of the three different appliances tested in the present study.

## DISCUSSION

This study compared the changes in soft profile on patients treated with Twin Block, Herbst mandibular propulsion appliances with dental anchorage or skeletal anchorage. All three protocols induced statistically significant changes in the soft face profile of the treated patients, especially in the mandibular measurements, which demonstrated protrusion of the lower lip and soft pogonium. However, no significant differences were detected between the effects obtained by the three protocols at the end of treatment. 

The results of this study corroborate those of the meta-analysis of Zymperdikas et al.,^17^ who evaluated the effects of fixed functional appliances on the treatment of Class II and detected a significant improvement in the profile of patients treated, with increase in mentolabial angle (+14.99% per year) and N-Sn-Pg (+2.0°/year), and a mild reduction of the H angle (-1.95°/year). In the present study, regarding the differences between the averages at T1 and T2, it was observed an increase of the mentolabial angle (HSA = 11.9°, HDA: 3.04°, TB: 5.16°), and a decrease in H angle (HSA: 3.27°; HDA: 1.64°; TB: 1.94°). In the three measures, the HSA group presented a more significant effect, although this difference was statistically non-significant. 

The present study also found different results from those reported by the systematic review of Flores-Mir et al.,^31^ which evaluated the soft profile changes observed in patients with Class II division 1 malocclusion treated with fixed functional appliances. Only five articles were included, of which four analyzed results from the Herbst appliances and one, from the Jasper Jumper. In this meta-analysis, an improvement of facial convexity and restriction of the anterior movement of the upper lip were observed; however, no change was noticed in the anteroposterior position of the lower lip and chin. In the present study, significant anterior displacement of the lower lip and the soft pogonium was detected.

The results of the present study are in accordance with the study conducted by Meyer et al.,^32^ which quantified three-dimensionally the effect of the Herbst appliance and the changes in the labial profile volume. They found a more significant development of the soft tissues of the mandible, with flattening of the curvature of the profile and anterior displacement of the mentolabial angle, with the most significant changes observed in the labial profile, with reduction of vermilion of the upper lip and increase of the height of the lower lip. In the present study, similar alterations were detected in all groups analyzed, with an increase in mentolabial angle and lower lip length.

The results found in this study were different from the study by Baysal and Uysal.^16^ While in the present study the lower lip and the soft pogonium displaced to anterior in all groups, the results were similar between the Herbst appliance with dental anchorage and the Twin Block, but were more pronounced in the Herbst appliance with skeletal anchorage; however, with no statistical significant difference between the groups. In the study by Baysal and Uysal ^16^, which compared the effects of Herbst and Twin Block appliances on the soft tissues of patients treated at the peak of the pubertal growth spurt, greater advancement of soft pogonium and lower lip was observed in patients treated with Twin Block than the ones treated with Herbst.

### LIMITATIONS

This study has some limitations. The first limitation was the exclusion of data from patients who abandoned treatment. The intention-to-treat analysis was not performed because the imputation of data in 40% of the sample could end up in biased information. Thus, the results of this study are valid only for patients who completed treatment, and the outcomes cannot be generalized for all patients. The second limitation was the unbalanced number of dropouts among the three groups. The higher number occurred in the HSA group (40%). The authors believe that this could be related to the discomfort caused by the skeletal anchorage in miniscrews. As a result, the outcomes from this group can likely change with the sample increase. Thus, this group should be analyzed with caution.

## CONCLUSION

Statistically significant changes were observed in the assessment of soft tissue measurements at the end of treatment when the appliances were evaluated individually, but they were not significant when the appliances were compared to each other.

The alterations favored the facial profile in Class II, division 1 patients treated by the three functional appliances studied (Twin Block, Herbst appliance with dental anchorage, and Herbst appliance with skeletal anchorage).

## Appendix 1: Definition of soft tissue measurements.

SOFT TISSUE ANGULAR MEASUREMENTS

• Convexity angle, including the nose: the angle formed between soft tissue nasion, nasal tip, and soft tissue pogonion.

• Convexity angle, excluding the nose: the angle formed between soft tissue nasion, subnasale, and soft tissue pogonion.

• Nasolabial angle: the angle formed between columella, subnasale, and soft tissue pogonion.

• Mentolabial angle: the angle formed between labrale inferioris, sulcus inferioris, and soft tissue pogonion.

• H angle: the angle formed between soft tissue nasion, soft tissue pogonion, and labrale superioris.

SOFT TISSUE LINEAR MEASUREMENTS

• Maxillary:

1. VRL-prn: horizontal distance between vertical reference line and pronasale.

2. VRL-sn: horizontal distance between vertical reference line and subnasale.

3. VRL-ss: horizontal distance between vertical reference line and sulcus superioris.

4. VRL-ls: horizontal distance between vertical reference line and labrale superioris.

5. E-ls: the distance from labrale superioris to a line joining the nasal tip and soft tissue pogonion.

6. Basic upper lip thickness: the dimension measured approximately 3 mm below point A and the drape of the upper lip.

7. Upper lip thickness: the dimension between the vermilion point and the labial surface of the maxillary incisor.

8. Upper lip strain measurement: the difference between the basic upper lip thickness and the upper lip thickness.

9. Upper lip length (Sn-uls): vertical distance between the upper lip stomion (uls) and the subnasale (Sn).

10. Interlabial gap: vertical distance between upper lip stomion (uls) and lower lip stomion (lls).

• Mandibular:

11. VRL-li: horizontal distance between vertical reference line and labrale inferioris.

12. VRL-si: horizontal distance between vertical reference line and sulcus inferioris.

13. VRL-pog: horizontal distance between vertical reference line and soft tissue pogonion.

14. E-li: the distance from labrale inferioris to a line joining the nasal tip and soft tissue pogonion.

15. Soft tissue chin thickness (Pog-pog): horizontal distance between hard and soft tissue pogonion.

16. Lower lip thickness at sulcus inferioris level (si-B): horizontal distance between sulcus inferioris and point B.

17. Lower lip thickness: horizontal distance between labrale inferioris and the most prominent point on buccal surface of lower incisor.

18. Lower lip length: vertical distance between lower lip stomion and soft tissue menton.
